# Sequence of 305,996 total hip and knee arthroplasties in patients undergoing operations on more than 1 joint

**DOI:** 10.1080/17453674.2019.1638177

**Published:** 2019-07-08

**Authors:** Peter Espinosa, Rüdiger J Weiss, Otto Robertsson, Johan Kärrholm

**Affiliations:** a Department of Molecular Medicine and Surgery, Section of Orthopaedics and Sports Medicine, Karolinska Institutet, Karolinska University Hospital, Stockholm;; b Swedish Knee Arthroplasty Register, Lund;; c Faculty of Medicine, Department of Clinical Sciences Lund, Orthopedics, Lund University, Lund;; d Swedish Hip Arthroplasty Register, Gothenburg;; e Department of Orthopaedics, Institute of Clinical Sciences, Sahlgrenska Academy, University of Gothenburg, Gothenburg, Sweden

## Abstract

Background and purpose — Patient-specific data on multiple total arthroplasties (TA) of the lower limbs due to osteoarthritis (OA) are limited. We investigated the sequence of surgical procedures and risk factors for additional surgery in such patients.

Patients and methods — 305,996 patients operated with a TA of the hip and/or knee due to OA were extracted from the Swedish National Hip (SHAR) and the Swedish Knee Arthroplasty Register (SKAR). 177,834 total hip arthroplasty (THA, 56% women, mean age 69 years) and 128,162 total knee arthroplasty (TKA, 60% women, mean age 69 years) procedures constituted the index operations. The mean, median, and maximum follow-up was 8, 6, and 23 years. Multivariable Cox regression analysis was used and Kaplan–Meier survival curves were constructed.

Results — Right-sided primary TA (34%) was most frequent. Subsequent surgery was most frequent after primary left-sided TKA (33%). The time interval to a second TA procedure was 3.1 (SD 3.2) years after TKA and 4.0 (SD 3.9) years after THA. After the index TA the probability of no subsequent surgery amounted to 64% (SD 0.3) for THA and 58% (SD 0.4) for TKA over 20 years. Lower age, female sex, left side, and TKA at index operation were associated with a higher probability for subsequent TA.

Interpretation — Delineation of factors that influence risk and the size of the risk for subsequent TA in 1 of the 3 major remaining joints is of value for clinicians and healthcare providers in the decision-making process for future resource allocation.

The progression of osteoarthritis (OA) is still unclear, particularly in patients with multiple severe OA in the large weight-bearing joints leading to total hip (THA) and total knee arthroplasty (TKA).

Previous studies suggest an association between the side of the first TA and the following THA or TKA (Shakoor et al. 2002, Gillam et al. 2013, Shao et al. 2013, Sanders et al. 2017). There are also a few other studies reporting on the outcome of patients with multiple TA (Papanikolaou et al. 2000, Mulhall et al. 2008, Hui et al. 2012). Some of these studies included relatively small patient numbers (< 100 patients) and were published more than a decade ago (Papanikolaou et al. 2000, Justen et al. 2003, Mulhall et al. 2008). We are only aware of a limited number of articles that have been population-based or published in the last decade (Gillam et al. 2012, 2013, Shao et al. 2013, Maradit Kremers et al. 2015, Sanders et al. 2017). Projections in Sweden show a steady increase to approximately 20,000 THA and 22,000 TKA performed in year 2030 which make TA one of the most common elective procedures (Nemes et al. 2014, 2015).

The projected increase in patients with multiple TA and the healthcare resources spent on this group of patients (Nemes et al. 2014, 2015) led us to investigate the sequence of surgical procedures and risk factors for additional surgery in patients with multiple TA of the lower limbs due to OA.

## Patients and methods

The Swedish Hip Arthroplasty Register (SHAR) is a national quality register that collects data on primary THA performed in Sweden since 1979. Revisions and reoperations are registered on an individual level. The completeness of the SHAR is around 98% (SHAR 2017). Since 1975 all primary and revision TKA performed in Sweden are gathered in the Swedish Knee Arthroplasty Register (SKAR). This register has national coverage and 97% completeness (SKAR 2018). Both registers record individual patient data such as age, sex, the diagnosis leading to surgery, side of the surgical procedure, surgical technique, and type of implant used. The diagnosis is reported to the SHAR and SKAR as found in patients’ medical records. No validation was performed for this study. SKAR started gathering detailed patient information on an individual level in 1975 and SHAR in 1992. At the time of the linkage of SHAR and SKAR for this study in 2016, data until 2014 were available.

**Figure 1.**
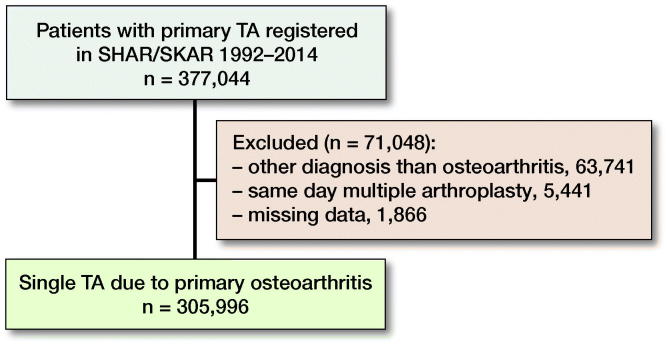
Flowchart showing patient selection. TA = total hip or knee arthroplasty, SHAR = Swedish Hip Arthroplasty Register, SKAR = Swedish Knee Arthroplasty Register.

By linking the registers, we could extract all patients operated with a THA and/or TKA during 1992–2014. This generated a data-set of 377,044 patients. After exclusions a final study cohort of 305,996 patients was formed (Figure 1). 

### Statistics

Demographic data are presented as means, standard deviations (SD), and ranges. We used Cox multiple proportional hazards regression analysis to evaluate factors with possible influence on the risk for subsequent TA surgery after initial TA. Hazard ratios (HR) with 95% confidence intervals (CI) are presented, both unadjusted and adjusted. Variables included in the Cox model were age, sex, side, type of TA, and time period for the index operation (1992–1998, 1999–2006 and 2007–2014). Subdivision of the time period was arbitrary. Proportionality was tested by plotting survival curves for the different variables individually and log-minus-log plots. Kaplan–Meier survival curves were constructed to evaluate the risk of a second TA over time. Patients were censored at their second TA, the end of the study period (December 31, 2014) or at death. Survival probabilities and 95% CIs are presented. Patients undergoing a 3rd and 4th single TA were comparatively few and are only presented as percentages. All analyses were performed using the PASW statistics package version 25 (SPSS Inc, Chicago, IL, USA).

### Ethics, funding, and potential conflicts of interest

Ethical approval was granted by the Regional Ethical Review Board in Gothenburg (approval number: 2017-01-26 dnr: 1034-16.). PE has been supported by grant from LINK Sweden AB. No competing interest declared.  

## Results

### Study population

177,834 patients underwent THA (56% women, n = 99,409) at a mean age of 69 (15–99) years. 128,162 patients underwent TKA (60% women, n = 76,641) at a mean age of 69 (12–97) years. The mean, median, and maximum follow-up was 8, 6, and 23 years, respectively. The data resulted in 40 different possible combinations of TA surgery sequences (Table 1, see Supplementary data).

### Distribution of patients with TA

Of all patients with a TA, 34% had a right-sided THA, 25% had a left-sided THA, 22% a right-sided TKA, and 19% a left-sided TKA as the first (index) surgical procedure. The sequence between index and subsequent TA are presented in Table 2. Table 3 (see Supplementary data) presents 3rd and 4th TAs after the index TA.

**Table 2.** Numbers at index operation and number of patients (%) who underwent subsequent joint replacements of the hip and/or the knee

First TA	No further			Second primary TA		
(index)	n	primary TA	THA right	THA left	TKA right	TKA left
THA right	102,705	76,618 (75)	–	21,568 (21)	1,643 (2)	2,876 (3)
THA left	75,129	53,868 (72)	18,106 (24)	–	2,329 (3)	826 (1)
TKA right	68,836	48,248 (70)	1,674 (2)	1,267 (2)	–	17,647 (26)
TKA left	59,326	40,069 (68)	1,594 (3)	988 (2)	16,675 (28)	–

**Figure 2.**
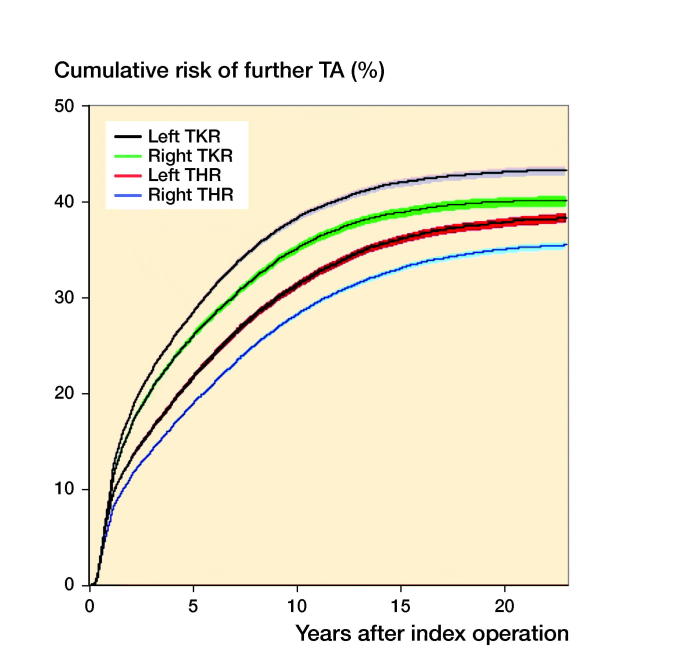
Cumulative (Kaplan–Meier) risk of further total joint arthroplasty (TA) after primary operation of right or left total hip arthroplasty (THA) or total knee arthroplasty (TKA).

The “survival” based on risk to be operated in 1 of the 3 remaining joints for patients alive during the study period was highest after right-sided index THA (65% at 22 years) followed by left-sided index THA (62%), index right (60%), and index left TKA (57%) (Figure 2).

### Cox regression analysis

The Cox regression analysis revealed that lower age (HR 1.028; CI 1.027–1.029), female sex (HR 1.14; CI 1.12–1.15), TKA at index operation (HR 1.33; CI 1.31–1.35), and left-sided index operation (HR 1.12; CI 1.10–1.13) were associated with an increased risk for at least 1 additional joint replacement of the hip or knee. The risk of undergoing at least 1 further TA was increased for each observation period (1999–2006: HR 1.23, CI 1.21–1.25; 2007–2014: HR 1.31, CI 1.28–1.33) (Table 4).

**Table 4.** Cox regression analysis: risk factors for further total joint arthroplasty (TA) of the hip and knee after the index TA during 1992–2014

Factor	Hazard ratio (95% CI)
Unadjusted	
Low age	1.028 (1.027–1.029)
Male sex	1.0 (ref.)
Female sex	1.08 (1.06–1.09)
Right side	1.0 (ref.)
Left side	1.14 (1.12–1.15)
THA	1.0
TKA	1.30 (1.28–1.32)
Year of surgery	
1992–1998	1.0 (ref.)
1999–2006	1.28 (1.26–1.30)
2007–2014	1.43 (1.40–1.46)
Adjusted^a^	
Low age	1.028 (1.027–1.029)
Male sex	1.0 (ref.)
Female sex	1.14 (1.12–1.15)
Right side	1.0 (ref.)
Left side	1.12 (1.10–1.13)
THA	1.0 (ref.)
TKA	1.33 (1.31–1.35)
Year of surgery	
1992–1998	1.0 (ref.)
1999–2006	1.23 (1.21–1.25)
2007–2014	1.31 (1.28–1.33)

a Adjustments for the following variables: age, sex, side, type of the TA and time period for the index operation.

### Time interval between index and subsequent TA

The mean time between surgery with an index THA and any further TA of the remaining 3 joints was 4 years (SD 4, range 0–23). The mean time after index TKA was shorter than after index THA (3 years, SD 3, range 0–22).

## Discussion

We found that patients had a slightly increased risk of at least 1 subsequent TA if they were younger, female, or if they had a left-sided index TKA. The probability to be operated in more than 1 joint increased during the study period. With the use of TA as a proxy for severe OA, we could confirm that the same joint on the contralateral side as the initial operation had the highest risk for further TA surgery. If the second operation after a THA involved the knee, this second operation was also most frequently localized to the contralateral side. Following initial TKA, the second operation, if a THA, was most commonly right-sided, disregarding localization of side for the index operation.

Gillam et al. (2013) found that more women than men had an index TA in the Norwegian population. This was also observed in our study. A higher proportion of subsequent right-sided rather than left-sided THA after an index TKA was found in the Norwegian population, as in our study. This similarity is not unexpected since Norway and Sweden share a border and the populations at large have a common genetic and cultural background. The higher probability for subsequent right-sided THA after index TKA in our study does not, however, completely concur with this previous study partly based on the Norwegian population. In that study based on both the Australian and Norwegian National Joint Registries, Gillam et al. (2013) found that patients primarily operated with TKA generally had a higher risk of receiving subsequent THA on the contralateral side.

Previous studies suggested that the progression of OA and TA surgery does not represent random progress (Shakoor et al. 2002, Gillam et al. 2013). We found a similar pattern as previously observed with a higher share of patients who had their subsequent TA in the cognate contralateral joint (Shakoor et al. 2002, Gillam et al. 2013, Shao et al. 2013, Sanders et al. 2017). We could also confirm the observation that patients who received a first-time THA and continued with a subsequent TKA were more frequently operated in the contralateral knee (Shakoor et al. 2002, Gillam et al. 2013, Sanders et al. 2017).

Sanders et al. (2017) described that younger age was a risk factor for subsequent THA after an index THA but not after an index TKA. This compares to a certain extent with our results where younger age was a risk factor for subsequent TA, but we did not perform separate analyses of index THA and TKA operations. Concerning the time interval between an index and a second TA, Shao et al. (2013) showed faster progress after an index TKA as observed in our study.

The degenerative joint disease in combination with pain may cause changes in the external muscular forces, the joint load, and the walking pattern, which in a complex way may influence the progression of the disease in other non-operated joints both before and after surgery (Shakoor et al. 2002). An indicator for this might be the progression from index TA to a subsequent TA of the same joint on the contralateral side. This may be explained by changes in weight load before and after surgery putting more stress on the remaining joints. Elimination of pain from 1 joint after insertion of a TA may also change the threshold for experience of pain from other joints with OA.

Localization and progression of the disease may also be related to the patient phenotype, suggesting that the pattern of OA evolution may vary depending on genetic background (Nelson et al. 2013). Some authors reported that willingness to undergo TA surgery may also vary between different patient groups and cultural backgrounds (Allen et al. 2014, Krupic et al. 2014).

We found an increased risk of multiple surgery during the later study period. The reason for this is not known, but increasing life expectancy, subtle changes of indication over time, increasing demands on an active lifestyle, and increased accessibility to health care may be possible explanations.

Our study has several limitations. Since this is a register-based study of TA surgery, only analyses of the sequence of severe OA requiring surgery could be studied, excluding all cases with OA not subjected to arthroplasty surgery. No radiographic analysis determining the severity of OA was performed. However, previous studies validated the use of TA as an appropriate indicator for severe OA (Shakoor et al. 2002, Dougados 2004, Lohmander et al. 2009). We included all patients operated with a primary THA and TKA registered in the SHAR and SKAR during 1992–2014. SHAR did not record data on patient identification before 1992 and therefore we cannot account for THA operations before 1992. SKAR has recorded data on patient identification since 1975 but for this study we did not exclude patients who received a TKA before 1992. Thus, some patients might have received a TA before 1992 although this subgroup of patients can be expected to be small due to a more conservative attitude towards TA surgery in Sweden during the years before 1992. A total of 1,866 incomplete surgical procedures were discarded. These patients represented only 0.6% of the total number but could nonetheless have an impact on the results and especially for patients with 3 and 4 TAs with comparatively few recorded procedures. Moreover, reoperations and revisions were not included in our study. Most probably, the occurrence of any type of reoperation may influence the willingness to undergo further surgery of other joints, though to what extent is unknown. Another factor to consider is surgeon’s preference and any bias associated with choice of 1st joint to operate in patients who at initial visit present with multiple joint severe OA. The diagnosis is reported to the SHAR and SKAR as found in patient medical records. No other validation was performed. There is always a risk of misclassification of the primary diagnosis, and especially in very young patients with joint disease classified as primary OA.

A strength is our large cohort of OA patients undergoing multiple TA of the hip and knee with a national coverage of almost 100%. This represents the first study where the SHAR and SKAR were merged on patient identification. To further explore the pattern of OA progression between different joints, studies including radiographic examination and non-operated cases would be necessary.

In summary, during an observation period of 20 years, we found that after TA due to primary OA the probability of no further TA in the remaining major joints of the lower extremity was about 60%, slightly higher after THA than after TKA. Lower age, female sex, left side, and TKA at index operation were associated with a higher probability for subsequent TA. If performed, the time interval to a second TA was almost 1 year shorter after index TKA than after index THA.

## Supplementary data

Tables 1 and 3 are available as supplementary data in the online version of this article, http://dx.doi.org/10.1080/17453674.2019.1638177

PE and JK: Conception and design of the study, analysis and interpretation of data, drafting the article and revision. RJW: Revision of article. OR: Analysis and interpretation of data, drafting the article and revision.  

*Acta* thanks Marianne Westberg for help with peer review of this study.

## Supplementary Material

Supplemental Material
