# Supplementary material for: Sequence of 305,996 total hip and knee arthroplasties in patients undergoing operations on more than 1 joint
Source: Acta Orthop. 2019 Jul 8;90(5):450–4. doi: 10.1080/17453674.2019.1638177 (PMC6746289; doi:10.1080/17453674.2019.1638177)
Supplement: Supplemental Material [file IORT_A_1638177_SM0352.pdf]

Supplementary data

Table 1. All patients (n = 377,044, all diagnoses) and all combinations and sequences of surgical procedures

Sequence	Patients (n)
H	174,794
K	95,100
H + H	44,659
K + K	34,926
H + K	7,030
K + H	5,644
KK	3,240
K + K + H	2,012
HH	1,738
H + H + K	1,683
H + K + K	1,282
H + K + H	1,015
K + H + H	911
K + H + K	814
H + H + K + K	463
K + K + H + H	405
KK + H	201
H + K + K + H	190
H + K + H + K	169
K + H + H + K	150
K + H + K + H	142
H + KK	130
HH + K	62
H + H + KK	59
KK + H + H	52
HK	48
K + HH	28
HH + K + K	23
HH + KK	23
K + K + HH	15
HK + K	7
K + HK	6
H + HK	5
KK + HH	5
HK + H	4
HK + HK	4
HK + K + H	2
H + HK + K	1
HK + H + K	1
K + H + HK	1

Table 3. Numbers at index operation and number (%) of patients who underwent subsequent 3rd or 4th total joint arthroplasty (TA) regardless of side

First TA (index)	n	3rd or 4th TA right or left
THA	177,834	889–2,845 (0.5–1.6)
TKA	128,162	641–2,563 (0.5–2.0)
